# The Synthetic Cannabinoid URB447 Reduces Brain Injury and the Associated White Matter Demyelination after Hypoxia-Ischemia in Neonatal Rats

**DOI:** 10.1021/acschemneuro.0c00047

**Published:** 2020-04-09

**Authors:** Silvia Carloni, Rita Crinelli, Linda Palma, Francisco J. Álvarez, Daniele Piomelli, Andrea Duranti, Walter Balduini, Daniel Alonso-Alconada

**Affiliations:** † Department of Biomolecular Sciences, University of Urbino Carlo Bo, Urbino, Italy; ‡ Biocruces Bizkaia Health Research Institute, Cruces University Hospital, Barakaldo, Bizkaia, Spain; § Departments of Anatomy and Neurobiology, Pharmaceutical Sciences, and Biological Chemistry, University of California, Irvine, Irvine, California, United States; ∥ Department of Cell Biology and Histology, School of Medicine and Nursing, University of the Basque Country (UPV/EHU), Leioa, Bizkaia, Spain

**Keywords:** Hypoxia-ischemia, cannabinoids, URB447, SR141716A, white matter demyelination, neuroprotection

## Abstract

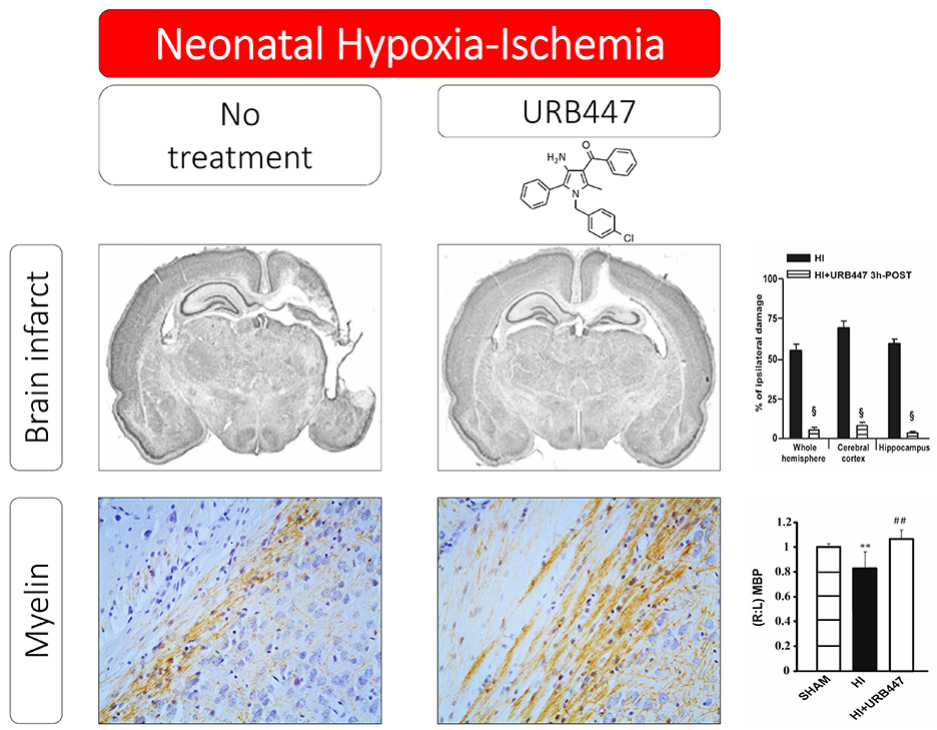

The number of functions controlled by the endocannabinoid system in health and disease continues growing over the years. In the brain, these include the modulation of harmful events such as glutamate excitotoxicity, oxidative stress, and inflammation, mainly regulated by activation/blockade of CB_1_/CB_2_ cannabinoid receptors. In the present work, we evaluated the capacity of the CB_1_ antagonist/CB_2_ agonist synthetic cannabinoid URB447 on reducing neurodegeneration after brain injury. By using a model of hypoxia-ischemia (HI) in neonatal rats, we found that URB447 strongly reduced brain injury when administered before HI. A comparable effect was observed with the CB_1_ antagonist SR141716A, whereas the CB_1_ agonist WIN-55,212-2 reduced the effect of URB447. When administered 3 h after HI, which is considered a clinically feasible therapeutic window to treat perinatal brain injury in humans, URB447 reduced neurodegeneration and white matter damage. Markers of astrogliosis and microglial activation also appeared reduced. These results confirm the important role played by the endocannabinoid system in the neurodegenerative process and strongly encourage further research into the mechanisms of URB447-induced neuroprotection.

## Introduction

The endogenous cannabinoid system is present throughout much of the body and is implicated in a variety of physiological functions, including feeding, modulation of pain, emotional behavior, and peripheral lipid metabolism.^1^ In the central nervous system (CNS), cannabinoids are able to limit the deleterious effects caused by multiple toxic stimuli, providing neuroprotection in different paradigms of brain injury. This is mainly obtained through their ability to modulate the intensity and extension of deleterious events, like glutamate excitotoxicity,^2−5^ nitric oxide and ROS production,^6^ and inflammation.^7,8^ Cannabinoids also regulate a wide number of beneficial processes, including induction of hypothermia^9^ and activation of cytoprotective signaling pathways, that control cell survival and fate.^10−12^ Because of these properties, it has been hypothesized that the endocannabinoid system might act as a natural neuroprotectant system.^13^ Therefore, compounds that modulate the endocannabinoid system could be promising neuroprotective agents.^14^

The classical way to modulate the endocannabinoid system is through cannabinoid (CB) receptors, namely, CB_1_ and CB_2_, which belong to the class A rhodopsin-like family of G-protein-coupled receptors. Their modulation can be achieved by exogenous administration of synthetic cannabinoids and phytocannabinoids or inhibitors of enzymes that degrade endocannabinoids, i.e., anandamide and 2-arachidonylglycerol. CB_1_ receptors are among the most abundant neuromodulatory receptors. They are primarily expressed in the brain and exhibit a presynaptic location.^15^ CB_2_ receptors, instead, are mainly expressed in tissues and cells of the immune system, including resident inflammatory cells within the CNS, and modulate the inflammatory response by decreasing the activity of antigen-presenting cells (APC) and down-regulating cytokine (IFN-γ and TNF-α) production,^8,16^ among others. Thus, a unique cannabinoid which interacts with CB_1_ and CB_2_ receptors or a combination of cannabinoids with different receptor selectivity could target a wide range of physiological conditions and change the progression of different neurological diseases. Indeed, cannabinoids could reduce excitotoxicity and nitric oxide and ROS production by acting through neuronal CB_1_ receptors, modulate reactive microgliosis by acting through microglial CB_2_ receptors, or activate cytoprotective pathways and enhance the trophic and metabolic support to neurons by acting through astroglial CB_1_ or CB_2_ receptors.

**Figure 1:**
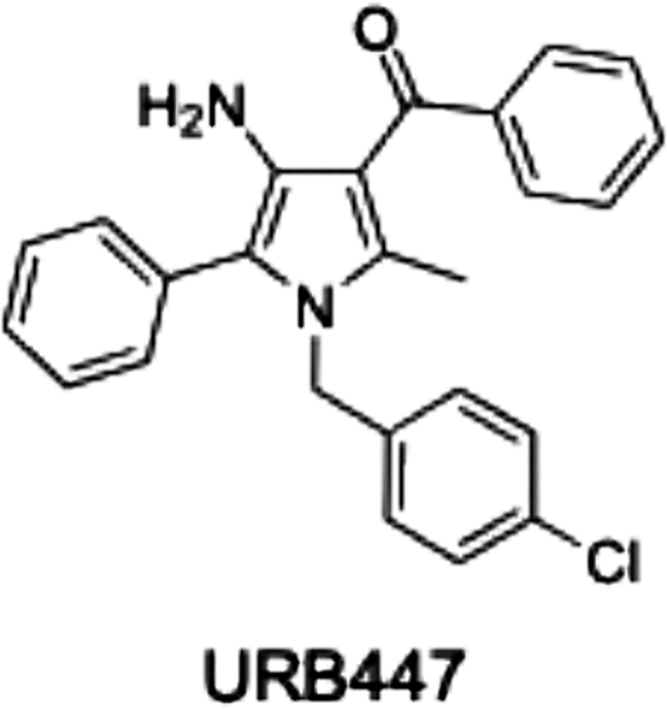
Chemical structure of URB447.

URB447 ({[4-amino-1-(4-chlorobenzyl)-2-methyl-5-phenyl-1*H*-pyrrole-3-yl](phenyl) methanone}) (Figure 1) is a synthetic CB receptor ligand based on a pyrrole scaffold variously substituted in all positions. It binds both CB_1_ and CB_2_ receptors with a submicromolar affinity and good stereoselectivity (CB_2_/CB_1_ ratio 9 < 10) and acts as a CB_1_ antagonist and CB_2_ agonist.^17^ Due to peripheral CB_1_ antagonism, URB447 was able to lower food intake and body-weight gain in mice,^17^ an effect also shown by the CB_1_ antagonist rimonabant (SR141716A).^18^ Interestingly, SR141716A also possesses neuroprotective effects in different models of brain injury, including NMDA-induced excitotoxicity in neonatal rats^19^ as well as permanent phototrombotic cerebral ischemia^20^ and permanent middle cerebral artery occlusion in adult rats.^21^ On the other hand, stimulation of CB_2_ receptors can dampen post-traumatic inflammation, as blockade or deletion of the CB_2_ receptors can worsen inflammation.^22^ Thus, we hypothesized that URB447, by possessing a mixed CB_1_ antagonist/CB_2_ agonist activity, could represent a putative candidate for reducing neurodegeneration after brain injury. To test this hypothesis, we used a neonatal rat model of hypoxia-ischemia (HI). Cannabinoid receptors and their endogenous ligands are present at relatively early stages of development and participate to the functional maturation of the CNS.^23^ Increased expression of CB receptors has been reported after immature brain injury.^24,25^

## Results and Discussion

### Pretreatment with URB447 and SR141716A Reduces HI-Induced Brain Damage

**Figure 2:**
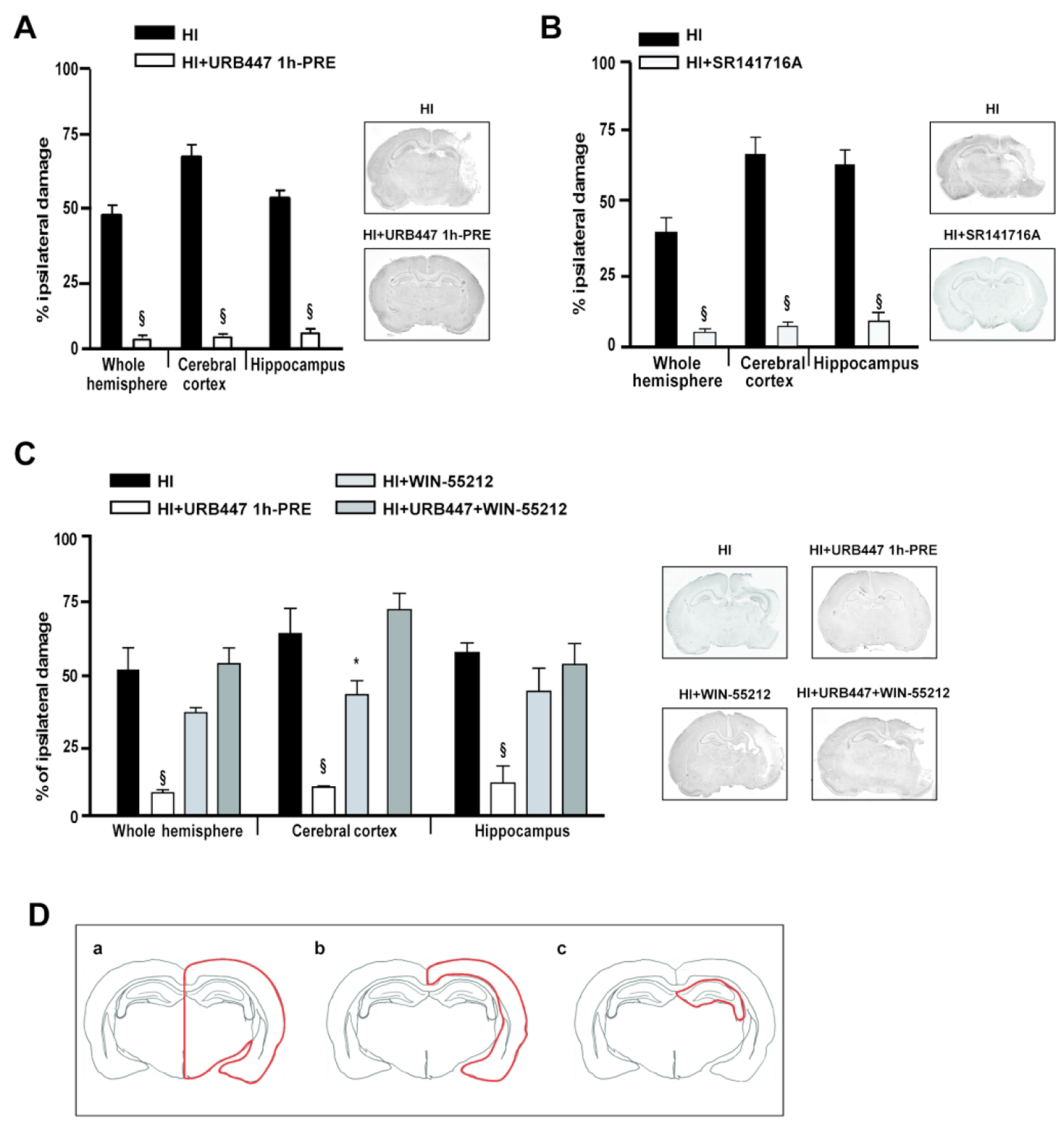
Evaluation of brain damage after neonatal hypoxia-ischemia (HI) and treatment with URB447, SR141716A, and WIN-55,212-2. Infarct volume measured in the whole hemisphere, cerebral cortex, and hippocampus of 14-day-old rats subjected to HI on P7 and treated with (A) vehicle (HI) or URB447 1 mg/kg 1 h before HI (HI+URB447 1h-PRE); (B) vehicle (HI) or SR141716A 1 mg/kg 1 h before HI (HI+SR141716A); (C) vehicle (HI) or URB447 1 mg/kg 1 h before HI (HI+URB447), or WIN-55,212-2 1 mg/kg 1 h and 30 min before HI (HI+WIN-55,212-2), or URB447 plus WIN-55,212-2 (HI+URB447+WIN-55,212-2). Images represent coronal brain sections at the hippocampal level of each experimental group stained with toluidine blue. Results are expressed as percentage of ipsilateral damage calculated from bilateral regional volumes using the formula 100(*L* – *R*)/*L*, where *L* is the volume of the contralateral region and *R* is the volume of the ipsilateral region (*N* = 10/group). * *P* < 0.05, § *P* < 0.001, Mann–Whitney test and one-way ANOVA followed by Newman–Keuls multiple comparison test. (D) Representative drawing of the brain areas—whole hemisphere (a), cerebral cortex (b), and hippocampus (c)—analyzed in the histological experiments reported in panels A, B, and C.

In adult animals, URB447 appeared restricted to the periphery,^17^ while there is no information if the compound passes the brain blood barrier (BBB) in neonates. Thus, we decided to perform pilot parallel experiments aimed at testing the effect of URB447 or SR141716A administered at the same dose before injury. In these experiments, we treated animals 1 h before HI and evaluated brain injury 7 days later. As shown in Figure 2A, the hypoxic-ischemic procedure induced a severe injury in the side of the brain ipsilateral to the occluded carotid, with the ipsilateral hemisphere showing a 46.5% reduction compared to the contralateral one. The most severe damage was observed in the cerebral cortex (65.7%; Figure 2A). Treatment with URB447 significantly reduced brain injury. The residual injury after URB447 was 16.3, 7.3, and 16.0% for the whole hemisphere, cerebral cortex, and hippocampus, respectively (Figure 2A). SR141716A also showed a robust neuroprotective effect. Residual injury after SR141716A administration was below 10% for the whole hemisphere and cortical and hippocampal brain regions (Figure 2B). Our findings agree with literature data showing that SR141716A can decrease infarct volume in transient and permanent middle cerebral artery occlusion models^26,27^ and suggest that blocking CB_1_ activation may be the main mechanism accounting for neuroprotection.

There is no information on URB447 pharmacokinetics and if it crosses the BBB in neonatal rats. In adult animals, however, this compound appears peripherally restricted.^17^ If we assume that URB447 holds the same features in adult and neonatal rats, it is possible to hypothesize that the slightly lower efficacy compared to SR141716A may reflect the higher capability of the latter to cross the BBB, which allows this compound to be present in the brain and rapidly block CB_1_ receptors in the early phase of injury. In contrast, during this early phase, URB447 could damp the peripheral inflammatory response and the recruitment of immune cells to the injury site by acting on CB_2_ receptors. This may reduce the inflammatory responses that contribute to CNS injury in the later phase.^28^ However, since after HI the BBB becomes permeable, it is realistic to predict that the strong neuroprotective effect could be due to both peripheral and central effects. The latter occurs through a direct agonist/antagonist CB_2_/CB_1_ receptor interaction on neuronal, glial, and microglial cells when URB447 enters in the CNS because of the leaky BBB. The strong neuroprotective effect observed after URB447 administration 30 min or 3 h after the hypoxic-ischemic procedure is in line with this hypothesis (see below). Moreover, when we tested the combined effect of URB447 and (*R*)-(+)-WIN-55,212-2 (WIN-55,212-2), a CB_1_/CB_2_-receptor agonist showing a higher preference toward CB_1_ receptors,^29^ we found that WIN-55,212-2 significantly reduced the neuroprotective effect of URB447 (Figure 2C). When WIN-55,212-2 was administered alone, instead, we found a slight reduction of brain injury (Figure 2C). This indicates that the simultaneous activation of both CB_1_ and CB_2_ receptors cannot result in maximal neuroprotection, as instead is obtained by combining CB_1_ inhibition with CB_2_ agonism.^30^ Conflicting results have been reported concerning the effect of WIN-55,212-2 in neuroprotection. For example, some authors reported neuroprotection in transient global ischemia and permanent focal ischemia in adult^31^ and neonatal rats.^32^ Others did not observe any decrease in either infarct volume or neurological outcome.^27^ The reason for these contrasting results as well as the role of activation of CB_2_ receptors by WIN 55,212-2 remains unclear, and the interaction of this compound with receptors different from CB receptors which may affect the outcome cannot be excluded.^33,34^ A recent work has shown a lack of protective effect of the selective CB_2_ receptor agonist GW405833,^35^ indicating that selective activation of CB_2_ receptors do not have the capacity to reduce infarct size or improve neurological outcomes after neonatal HI, despite the several doses and administration regimens tested, adding complexity to the effect of cannabinoid interacting compounds in neurodegeneration.

### Post-Treatment with URB447 Reduces HI-Induced Brain Damage, White Matter Demyelination, Astrogliosis, and Microglial Activation

**Figure 3:**
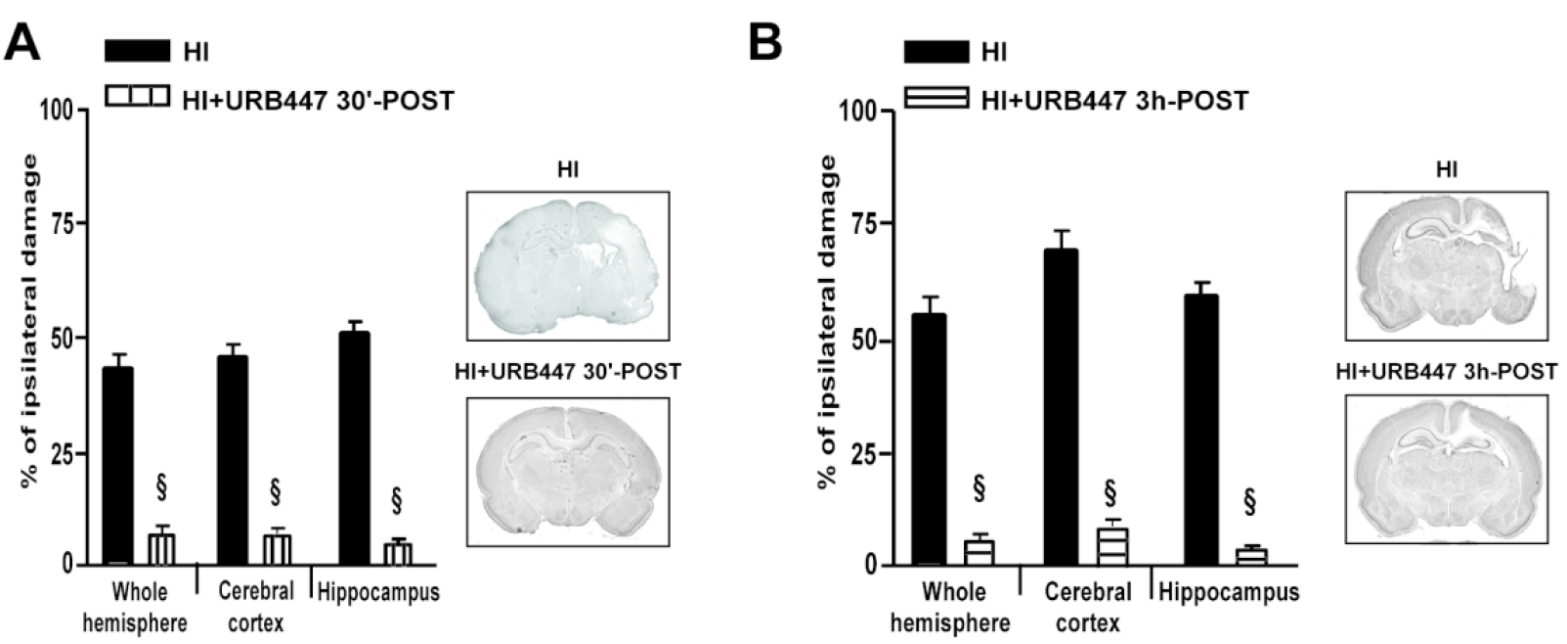
Evaluation of brain damage after neonatal hypoxia-ischemia (HI) and treatment with URB447. Infarct volume measured in the whole hemisphere, cerebral cortex, and hippocampus of 14-day-old rats subjected to HI on P7 and treated with (HI) or URB447 1 mg/kg 30 min after HI (HI+URB447 30 min-POST) (A), or URB447 1 mg/kg 3 h after HI (HI+URB447 3h-POST) (B). Images represent coronal brain sections at the hippocampal level of each experimental group stained with toluidine blue. Results are expressed as percentage of ipsilateral damage calculated from bilateral regional volumes using the formula 100(*L* – *R*)/*L*, where *L* is the volume of the contralateral region and *R* the volume of the ipsilateral region (*N* = 10/group). § *P* < 0.001, Mann–Whitney test.

**Figure 4:**
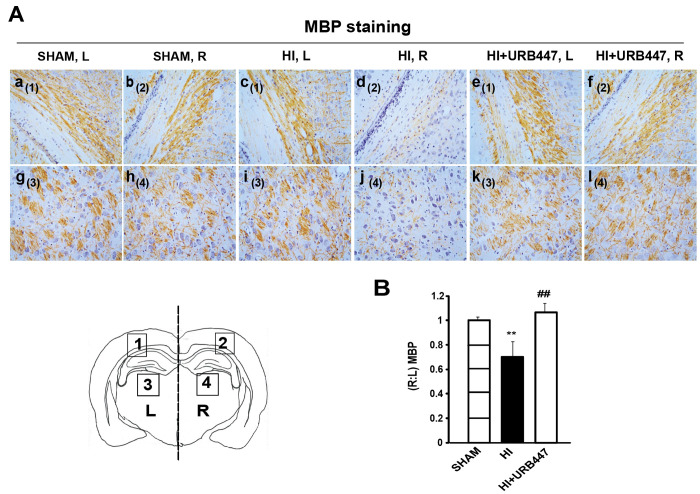
Modulation of myelin basic protein (MBP) staining by neonatal hypoxia-ischemia (HI) and treatment with URB447 in the mid-dorsal hippocampus. (A) Light microphotographs illustrating the disruption of MBP immunostaining in external capsule (a–f) and adjacent striatum (g–l). Samples were obtained from 14-day-old rats subjected to sham operation (SHAM; a, b, g, h) or to HI on P7 and treated with either vehicle (HI; c, d, i, j) or URB447 (HI+URB447; e, f, k, l). (B) Densitometric analysis performed as described in the Materials and Methods section. L, left side, contralateral; R, right side, ipsilateral to occluded carotid artery. Bars, 100 μm. ** *P* < 0.01 vs Sham group, ## *P* < 0.01 vs HI group, one-way ANOVA (*N* = 8/group).

**Figure 5:**
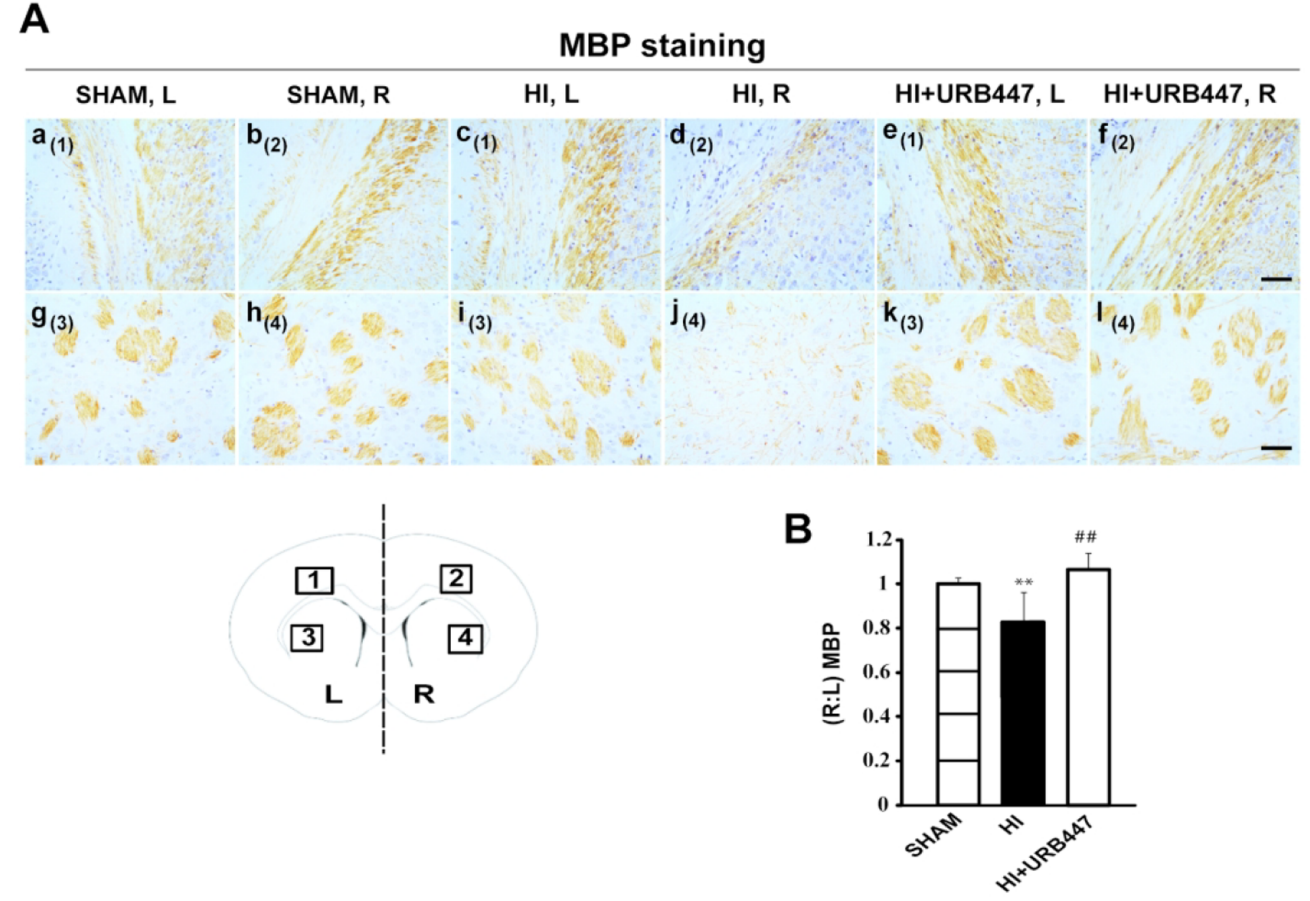
Modulation of myelin basic protein (MBP) staining by neonatal hypoxia-ischemia (HI) and treatment with URB447 in the striatum. (A) Light microphotographs illustrating the disruption of MBP immunostaining in the external capsule (a–f) and adjacent striatum (g–l). Samples were obtained from 14-day-old rats subjected to sham operation (SHAM; **a**, b, g, h) or to HI on P7 and treated with either vehicle (HI; c, d, i, j) or URB447 (HI+URB447; e, f, k, l). (B) Densitometric analysis performed as described in the Materials and Methods section. L, left side, contralateral; R, right side, ipsilateral to occluded carotid artery. Bar, 100 μm. ** *P* < 0.01 vs Sham group, ## *P* < 0.01 vs HI group, one-way ANOVA (*N* = 8/group).

To assess further the neuroprotective effect of URB447, we performed experiments by administering the compound 30 min or 3 h after the initial insult. Postinjury administration of URB447 does not have the problem of BBB crossing, since injury allows the leakage of the BBB^36^ with a better penetration of the compound into the CNS. As shown in Figure 3, residual injury in the HI+URB447 30 min-POST and in the HI+URB447 3h-POST groups was, respectively, 4.3 and 12.0% for the whole hemisphere, 4.6 and 15.4% for the cerebral cortex, and 8.1 and 11.2% for the hippocampus (Figure 3A and B). Since a pharmacological intervention within 3 h after the injury is considered a clinically feasible therapeutic window to treat perinatal brain injury in humans,^37^ we decided to better characterize the effect of URB447 administered at this time point, focusing on the consequences of HI and URB447 administration on white matter injury and activation of glial cells. Indeed, white matter is particularly susceptible to perinatal asphyxia, and cerebral white matter injury is a common feature of hypoxic-ischemic encephalopathy.^38,39^ Studies in human and rodent models have described many histological features in the white matter that correlate to early^40^ and late^41^ cognitive impairment in children; these include cell death, edema, gliosis, and reduced myelination.^41^Figure 4A shows myelin basic protein (MBP) immunostaining performed 7 days after HI at the level of the mid-dorsal hippocampus and thalamus. Hypoxic-ischemic animals showed a substantial loss of ipsilateral MBP immunostaining external capsule (Figure 4A, panel d) and adjacent striatum (Figure 4A, panel j), indicative of the marked loss of axonal processes. A similar effect was observed at the level of the mid-striatum (Figure 5), which also displayed a substantial loss of ipsilateral MBP immunostaining in the external capsule (Figure 5A, panel d) and adjacent striatum (Figure 5A, panel j) when compared with sham animals (Figure 5A, panels b and h). The reduction in the immunostaining pattern of MBP after HI can be easily observed when comparing the ipsilateral regions with the contralateral ones of HI and sham animals (HI, Figure 4A, panels c and i, and Figure 5A, panels c and i; sham, Figure 4A, panels a and g, and Figure 5A, panels a and g). URB447-treated animals displayed a similar staining pattern (Figure 4A, panels e, f, k, and l, and Figure 5A, panels e, f, k, and l) to sham animals. Densitometry analysis shows that HI caused a significant loss in MBP immunostaining (*P* < 0.01) in the subcortical white matter and striatum both at the level of mid-dorsal hippocampus and thalamus (0.69 ± 0.13; Figure 4B) and mid-striatum (0.81 ± 0.08; Figure 5B). The decrease in the (*R*:*L*) MBP ratio was absent in URB447-treated rats in both anatomical regions (1.07 ± 0.07 and 1.04 ± 0.04, respectively; Figures 4B and 5B). In this group of animals, values were similar to those observed in the sham group. A similar effect was previously found after administration of melatonin as a neuroprotectant,^42^ indicating that myelination disturbances in the external capsule, cingulum (with processes extending into the adjacent cortex), and striatum can be attenuated independently of the type of neuroprotective agent.

**Figure 6:**
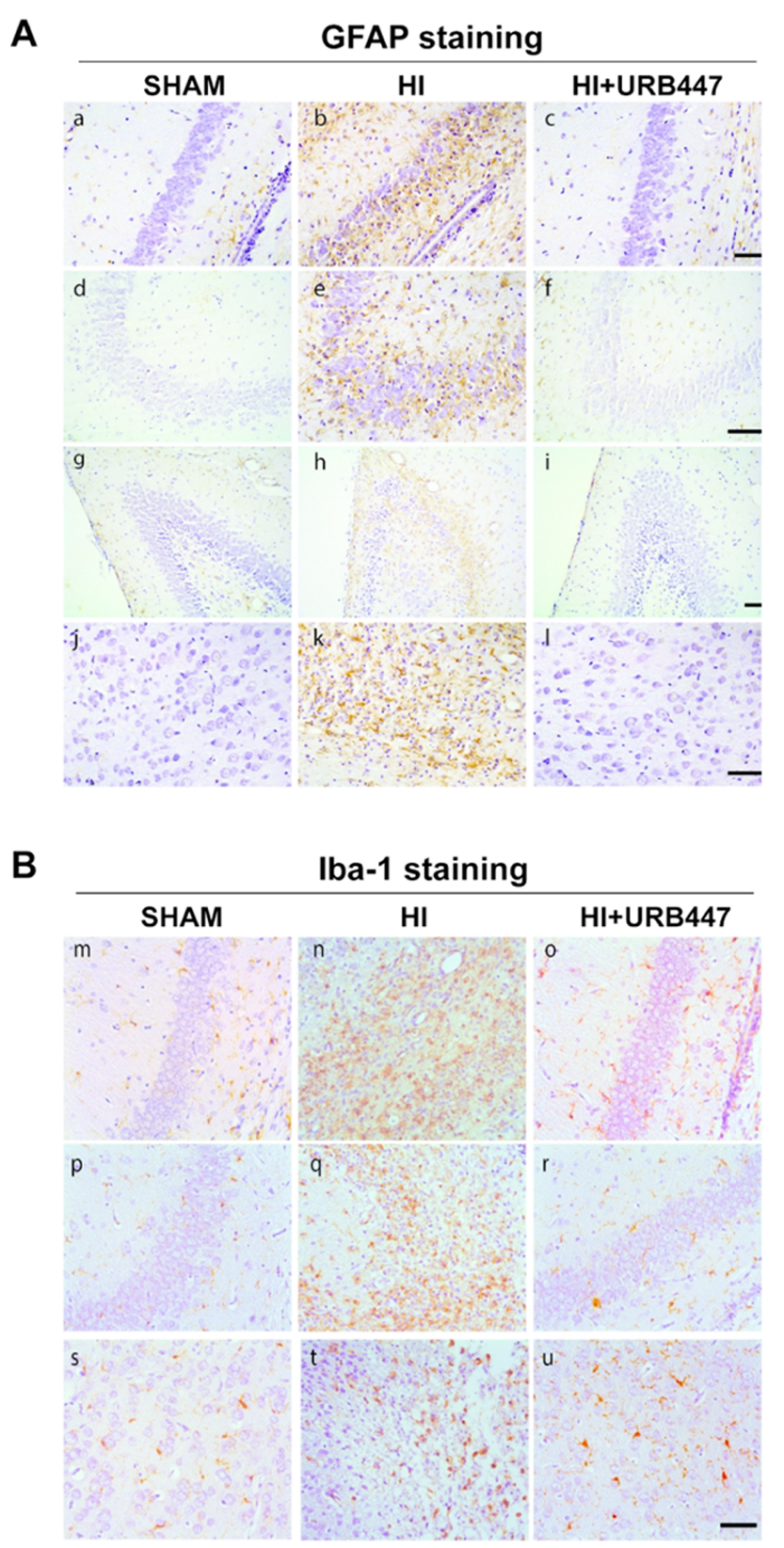
Modulation of glial fibrillary acidic protein (GFAP) and ionized calcium binding adaptor molecule-1 (Iba-1) staining by neonatal hypoxia-ischemia (HI) and treatment with URB447. (A) Representative light microphotographs illustrating GFAP immunostaining in brain sections from CA1 (a–c) and CA2/CA3 (d–f) hippocampal regions, dentate gyrus (g–i) and cortex (j–l). Samples were obtained from 14-day-old rats subjected to sham operation (SHAM; a, d, g, j) or to HI on P7 and treated with either vehicle (HI; b, e, h, k) or URB447 (HI+URB447; c, f, i, l). (B) Representative light microphotographs illustrating Iba-1 immunostaining in brain sections from CA1 (m–o) and CA2/CA3 (p–r) hippocampal regions and cortex (s–u). Samples were obtained from 14-day-old rats subjected to sham operation (SHAM; m, n, o) or to HI on P7 and treated with either vehicle (HI; n, q, t) or URB447 (HI+URB447; o, r, u). Bars, 100 μm.

To study the outcome in white matter elements, we evaluated the glial fibrillary acidic protein (GFAP, an intermediate filament protein in astrocytes) and the ionized calcium binding adaptor molecule-1 (Iba-1, a microglia/macrophage-specific calcium-binding protein) immunostaining. Astrocyte reactivity was prominent in the ipsilateral hemisphere of HI animals, with GFAP-positive cells surrounding necrotic-like cells at the level of CA1 (Figure 6A, panel b) and CA2/CA3 areas of the hippocampus (Figure 6A, panel e), dentate gyrus (Figure 6A, panel h), and cortex (Figure 6A, panel k). In contrast, low levels of astrogliosis were detected in both sham (Figure 6A, panels a, d, g, and j) or URB447-treated animals (Figure 6A, panels c, f, i, and l). When evaluating Iba-1, HI resulted in the loss of the microglial branches, with many cells transforming into completely rounded brain macrophages (Figure 6B, panels n, q, and t), an effect evident in the core area of the infarct. The expression pattern of Iba-1 was similar to that observed for GFAP, with sparse reactivity in the hippocampus and cortex from sham pups (Figure 6B, panels m, p, and s) or animals receiving URB447 (Figure 6B, panels o, r, and u). Astrocytes apparently increased in number and showed longer cytoplasmic processes, whereas microglial cells exhibited a rounded phenotype related to their activated phagocytic activity. In the early response to brain injury, astrocytes participate in the formation of the so-called glial scar, which serves to stop excessive cell extravasation from damaged blood vessels. Together with cellular morphological changes, a common feature of astrogliosis is the increased expression and aggregation of astrocytic cytoskeleton proteins like the GFAP.^43^ Reactive astrogliosis may persist for months or even years after injury, inducing neuronal signaling impairment and blocking axonal regrowth and remyelination.^44,45^ During the evolving damage, overactivation of CNS-infiltrating macrophages derived from circulating monocytes and microglia can be either beneficial or detrimental for the injured brain. The dual capacity of microglia to facilitate regeneration and repair or to exacerbate cerebral damage depends on multiple factors but appears related to its phenotype. Rounded amoeboid-like microglial cells can release a wide variety of substances linked to the neuroinflammation process, comprising nitric oxide,^46^ pro-inflammatory cytokines,^47^ and reactive oxygen species.^48^ These inflammatory mediators can in turn promote leukocyte diapedesis into the injured brain parenchyma and/or induce direct neurotoxicity and subsequent cell death, thereby contributing to evolving neuronal and white matter injury.^49^ In URB447-treated rats, both GFAP and Iba-1 immunostaining patterns appeared similar to those observed in control animals, indicating that URB447 reduced reactive astrogliosis and microglial activation, key players in neuroinflammation and myelination deficiencies.^44^ Inhibition of reactive astrogliosis and microglial activation may be, therefore, a significant contributor for cell survival and reduction of local tissue damage obtained with cannabinoid-related substances,^50^ including URB447, or other therapies such as hypothermia.^51,52^

## Conclusions

Accumulating data indicate that the differential modulation of the endocannabinoid system by means of CB_1_ receptor blockage and/or CB_2_ receptor stimulation can exert protective responses in different experimental paradigms. In ischemic brain injury, Zhang and colleagues^53^ reported that administration of the CB_1_ antagonist SR141716A together with the CB_2_ agonist O-1966 exerted a stronger effect in reducing cerebral infarction than the administration of the single molecules. In chronic liver damage, CB_1_ receptor antagonism^54,55^ as well as the activation of CB_2_ receptors^56^ can ameliorate liver fibrosis. In human macrophages, the possibility of blocking CB_1_ receptors in combination with selective activation of CB_2_ receptors has been suggested to reduce inflammatory responses, as the first upregulates both reactive oxygen species and cytokine expression while the latter reduces CB_1_-stimulated ROS production.^57^ Here we demonstrate a robust neuroprotective effect achieved with the CB_1_ antagonist/CB_2_ agonist URB447. Neuroprotection was observed even when URB447 was administered 3 h after the initial insult, which is considered a clinically feasible therapeutic window to treat perinatal brain injury in humans.^37^ We believe that this compound deserves further studies to better address the mechanism(s) of its robust neuroprotective effect.

## Materials and Methods

### Cerebral HI

All surgical and experimental procedures were carried out in accordance with the Italian regulation for the care and use of laboratory animals (Directive 86/609/EEC) and were approved by the Animal Care Committee of the University of Urbino Carlo Bo. Pregnant Sprague–Dawley rats were housed in individual cages, and the day of delivery was considered day 0. Neonate rats from different litters were randomized, normalized to 10 pups per litter, and kept in regular light/dark cycle (lights on 8 am to 8 pm). On postnatal day 7 (P7), after anesthesia with 5% isoflurane in N_2_O/O_2_ (70/30) mixture, rat pups underwent unilateral ligation of the right common carotid artery via a midline neck incision. After artery ligation, the wound was sutured and the animals allowed to recover for 3 h under a heating lamp. Pups were then placed in an airtight jar and exposed for 2.5 h to a humidified nitrogen–oxygen mixture (92 and 8%, respectively) delivered at 5–6 L/min (HI). The jar was partially submerged in a 37 °C water bath to maintain a constant thermal environment.

### Drug Supply and Dosage

URB447 was synthesized as previously described.^17^ SR141716A and WIN-55,212-2 were purchased from Sigma-Aldrich. Drugs were dissolved in 1:9 PBS:DMSO (vehicle) and injected intraperitoneally to a final concentration of 1 mg/kg. This dose was chosen based on what was previously reported in the literature for the CB_1_ antagonist SR141716A^21^ and WIN-55,212-2,^19^ and on preliminary experiments performed with URB447 and SR141716A administered before the HI induction.

### Experimental Groups and Treatments

Initially, animals were treated 1 h before the ischemic/hypoxic procedure with a single intraperitoneal (IP) injection of URB447 (HI+URB447 1h-PRE, *N* = 10) or with the CB_1_ antagonist SR141716A (HI+SR141716A, *N* = 10). WIN-55,212-2 or the corresponding volume of vehicle were injected 1 h and 30 min before HI to the HI+WIN-55,212-2 and HI+URB447+WIN-55,212-2 groups (*N* = 10/group). The HI-injured groups (HI, *N* = 10/group) received a corresponding volume of vehicle. Afterward, the neuroprotective effect of URB447 was further assessed by administering it after HI. We used two different groups of animals treated 30 min after hypoxia-ischemia (HI+URB447 30 min-POST, *N* = 10) or 3 h after hypoxia-ischemia (HI+URB447 3h-POST, *N* = 10) with a single IP injection of URB447. The HI groups (*N* = 10/group) received a corresponding volume of vehicle. Brain histology was performed on additional groups of vehicle-treated (sham, *N* = 8), HI (*N* = 8), and HI+URB447 3h-POST (*N* = 8) animals.

### Brain Injury Assessment

On P14, animals were deeply anesthetized and perfusion-fixed with 4% paraformaldehyde (PFA) in 0.1 mol/L PBS. Brains were rapidly removed on ice, immersion-fixed in 4% PFA at 4 °C for 4 h, and cryoprotected with 8% sucrose in PBS (72 h, 4 °C). To evaluate tissue injury, coronal sections of the brain of each animal (40 μm thick) were cut on a cryostat and thaw-mounted onto acid-washed subbed slides (gelatin and chrome alum). Sections were then stained with toluidine blue. A computerized video camera-based image analysis system (ImageJ 1.45 software; https://imagej.nih.gov/ij/) was used to measure cross-sectional areas from the level of the anterior genu of the corpus callosum to the end of the gyrus dentatus. Measurements, based on the intensity and uniformity of the staining, were performed by an experimenter that was blinded to the conditions of the treatment and included only intact tissue. Regional volumes were estimated by summing areas and multiplying by the distance between sections (40 μm). Percent reduction in whole hemisphere or in the selected brain regions was calculated by using the formula 100 × (left side volume – right side volume)/left side volume.

### Brain Histology

#### Tissue Collection

On PN14, pup rats were deeply anesthetized with 5% isoflurane in N_2_O/O_2_ (70/30%) mixture and perfusion-fixed with 4% PFA in 0.1 μM PBS. Brains were removed, immersed in the same fixative at 4 °C overnight, and embedded in paraffin. Brains were sectioned at a thickness of 5 μm at stereotaxic standard levels of 1.6 (1.6 mm anterior to the interaural line per Sherwood and Timiras, 1970), 2.0, and 2.3 A, at the level of mid-dorsal hippocampus and thalamus. Brain sections were collected using polylysine-coated slides and processed for immunohistochemistry and white matter injury analysis.

#### Immunohistochemistry (IHC)

Sections were rehydrated and endogenous peroxidase blocked with 1% H_2_O_2_, then with appropriate serum together with 0.1 Triton in PBS and probed overnight at 4 °C with the following primary antibodies: anti-GFAP (1:300, Dako, Denmark), anti-Iba1 (1:1000; Wako, Osaka, Japan), or anti-MBP (1:200, Santa Cruz Biotechnology, CA, USA). Sections were incubated with peroxidase-labeled secondary antibody (1:100, Santa Cruz Biotechnology, CA, USA) for 1 h, and staining was visualized using diaminobenzidine and counterstained with hematoxylin. Then, sections were dehydrated and coverslipped with DPX (VWR, Leighton Buzzard, U.K.).

#### White Matter Injury Assessment

The MBP expression pattern was evaluated by densitometry in order to analyze white matter injury. As previously described,^58^ the measurement of the MBP immunostaining pattern was carried out using brain images obtained at the level of the mid-striatum and at mid-dorsal hippocampus. Specifically, six sections per brain, three at the level of the mid-striatum and three at the mid-dorsal hippocampus, were evaluated. Images were digitized, segmented (using a consistent arbitrary threshold of −50%), and binarized (black vs white) using a computerized video-camera-based image-analysis system (ImageJ 1.45 software; https://imagej.nih.gov/ij/). Total black pixels per hemisphere were counted, and average values were calculated per brain, expressed as pixels per hemisphere. Densitometric values were expressed as ratios of right-to-left hemispheric measurements as follows: for each brain sample, (*R*:*L*)MBP of pixels per left hemisphere to pixels per right hemisphere was calculated. At least five sections per brain were analyzed, and only sections with obvious technical artifacts related to the staining procedure were excluded.

### Statistical Analyses

Statistical analyses were performed using the Prism Computer program (graphpad.com). In vivo experimental data were analyzed by the Mann–Whitney test or one-way ANOVA followed by Newman–Keuls multiple comparison test. Values are presented as mean ± SEM (in vivo experiments) and were considered significant when *P* ≤ 0.05.
